# Awareness of Vision Zero among United States’ road safety professionals

**DOI:** 10.1186/s40621-018-0151-1

**Published:** 2018-05-08

**Authors:** Kelly R. Evenson, Seth LaJeunesse, Stephen Heiny

**Affiliations:** 1 Department of Epidemiology, Gillings School of Global Public Health, University of North Carolina – Chapel Hill, Chapel Hill, North Carolina USA; 2 Highway Safety Research Center, University of North Carolina at Chapel Hill, Chapel Hill, North Carolina USA

**Keywords:** Awareness, Bicycling, Diffusion of innovations, Motor vehicle crashes, Road safety, Pedestrians, Walking

## Abstract

**Background:**

Vision Zero is a strategy to eliminate all fatalities and serious injuries from road traffic crashes, while increasing safe and equitable mobility for all. In 2015, the United States’ Department of Transportation announced the official target of the federal government transportation safety policy was zero deaths. In 2017, we assessed the dissemination of Vision Zero in the United States.

**Methods:**

We conducted a web-based survey in 2017 among road safety professionals. Email invitations were sent using relevant membership directories and conference lists.

**Results:**

We surveyed 192 road safety professionals, including planning/engineering (57.8%), public health (16.7%), and law enforcement/emergency medical services (EMS) (8.9%). Awareness of Vision Zero was higher among planning/engineering fields (97.3%) compared to law enforcement/EMS (76.5%) and public health (75.0%). Awareness was similar by number of years working in the field. Awareness was higher in the South (95.9%) and Northeast (95.0%) regions, followed by the West (90.8%) and Midwest (85.2%) Census regions. Among those that heard of Vision Zero (*n* = 174), 41.8% worked at a municipality with a Vision Zero campaign, while 41.2% did not. Among those working at a municipality with a Vision Zero campaign (*n* = 71), about half participated in the campaign (54.9%) while the other half did not (45.1%).

**Conclusions:**

With widespread dissemination of the Vision Zero strategy to road safety professionals, next steps include evaluating how Vision Zero is being adopted, implemented, and maintained in communities, as well as the awareness and acceptability by community members, and to identify the most promising policies and practices.

**Electronic supplementary material:**

The online version of this article (10.1186/s40621-018-0151-1) contains supplementary material, which is available to authorized users.

## Background

In the United States, in 2016 there were 34,439 fatal motor vehicle crashes in which 37,461 deaths occurred, resulting in 1.2 deaths per 100 million miles traveled (Insurance Institute for Highway Safety, Highway Loss Data Institute, 2018). The United States’ crash death rate is more than twice the average of countries such as Australia, Canada, Israel, Japan, New Zealand, Norway, Switzerland, and countries in the European Union (Ahangari et al., 2016; Centers for Disease Control and Prevention, National Center for Injury Prevention and Control, 2016). In the United States, in 2015 there were 5376 pedestrians and 818 bicyclists/cyclists killed in traffic crashes, a 9.5% and 12.2% increase from the prior year, respectively (United States Department of Transportation, National Highway Traffic Safety Administration, 2017a, b). Pedestrians comprised 15.3% of all traffic fatalities and approximately 2.9% of all traffic-related injuries (United States Department of Transportation, National Highway Traffic Safety Administration, 2017b), while bicyclists/cyclists comprised 2.3% of all traffic fatalities and approximately 1.8% of all traffic-related injuries (United States Department of Transportation, National Highway Traffic Safety Administration, 2017a).

One way to address the unacceptable burden of motor vehicle crashes and associated injuries and deaths is through a project called Vision Zero. Vision Zero takes a systems perspective to reducing fatalities and serious injuries from road traffic crashes to zero, while increasing equitable, safe, and healthier mobility for all (Kim et al., 2017; Vision Zero Network, 2017). It focuses on decreasing the likelihood that crashes will result in serious injury or death by designing safer transportation systems (Fleisher et al., 2016). Vision Zero can change a once held predominant perspective, that traffic deaths are inevitable and an individual’s responsibility, to instead a perspective that traffic deaths are preventable and requires a system approach (Vision Zero Network, 2017). The Vision Zero Network provides tools to guide education, design, structural improvements, and enforcement (Vision Zero Network, 2017). The program recognizes there is no one right way towards implementation of Vision Zero (Kim et al., 2017).

In 1997, the Swedish Parliament (Sweden’s highest decision-making body) adopted Vision Zero (Belin et al., 2012; Fahlquist, 2006). Since then, the vision has spread worldwide. For example in the United States, in 2010 the state of Washington launched Target Zero (Thomas et al., 2015), and in 2012 the Seattle Department of Transportation launched a similar plan (Seattle Department of Transportation, 2012), both to eliminate serious and fatal crashes by the year 2030. In January 2015, the United States’ Department of Transportation announced that the official target of the federal government transportation safety policy was zero deaths. A year later (January 2016), ten United States’ cities collectively announced plans to lead initiatives to eliminate traffic fatalities on their roadway networks (Shahum, 2016). These cities included: Austin, Texas; Boston, Massachusetts; Chicago, Illinois; Fort Lauderdale, Florida; Los Angeles, California; New York City, New York; Portland, Oregon; San Francisco, California; Seattle, Washington; and Washington, D.C.

Despite these recent announcements at federal and municipal levels, the extent of dissemination of Vision Zero among United States’ road safety professionals. One of the keys to an effective Vision Zero commitment is to have involvement from multi-disciplinary leadership, cooperation, and collaboration among road safety professionals (Vision Zero Network, 2017), including those involved in planning, engineering, public health, law enforcement, and emergency medical services (EMS). Thus, our overarching goal was to discover the current dissemination of Vision Zero among road safety professionals through their awareness and any implementation in their local community. We also explored whether awareness of Vision Zero varied by road safety professionals’ field of work, time spent in the field, or location.

## Methods

### Sample

The survey sample consisted of road safety professionals who may be involved with Vision Zero, including planning, engineering, public health, law enforcement, and EMS. Membership directories (American Public Health Association, Association of Pedestrian and Bicycle Professionals, Transportation Research Board committees) and conference lists (Lifesavers National Conference on Highway Safety Priorities) were used to identify safety-focused professionals. All professionals listed in these directories with an email were invited to participate in the survey.

### Survey and analysis

The survey questions were guided by the Diffusion of Innovations theory (Rogers, 2003). The theory can help explain how an idea (i.e., Vision Zero) spreads or diffuses into the population (i.e., safety professionals). The survey was developed, piloted, and revised before administering it using a Qualtrics (Provo, Utah and Seattle, Washington) platform between June 20, 2017 to July 10, 2017 via web or mobile application. This project was reviewed and exempted by the Institutional Review Board at the University of North Carolina. The initial survey screen detailed a consent form. Following this form, respondents were asked whether or not they would be participating in the survey. Those that answered affirmatively were then asked a screening question: “Does your work involve understanding or improving the safety of people on roadways?” If the answer was “yes”, then they were prompted with the rest of the survey (Additional file 1).

Participants were asked if they had heard of Vision Zero, “a municipality-led strategy to eliminate all traffic fatalities and severe injuries, while increasing safe, healthy, equitable mobility for all”. If “yes”, then they were prompted to respond to what year they learned of it, whether their municipality had a campaign, and if they were involved in the campaign. The survey asked all participants to identify their field of work, length of time in the field, and the name and location of their work organization. Based on work location, states were assigned to a 2010 census region (https://www.census.gov/geo/reference/gtc/gtc_census_divreg.html). Differences in Vision Zero awareness were explored using Pearson’s chi-squared tests (Pagano and Gauvreau, 2000). Survey data were imported and analyzed in SAS version 9.3 (Cary, North Carolina).

## Results

In total, 1738 professionals were contacted by email (957 planning/engineering, 516 public health, 265 law enforcement/EMS) from each of the four census regions (323 Midwest, 265 Northeast, 624 South, and 526 West). We included surveys if the question on awareness of Vision Zero was answered (*n* = 192). Response rates could be approximated based on participant’s field of study (11.6% (111/957) planning/engineering, 6.4% (17/265) law enforcement/EMS, 6.2% (32/516) public health) (response rates are slightly higher than what is calculated given *n* = 32 participants with “Other” fields of work and emails that were not read). Response rates were similar by census region (8.4% (27/323) Midwest, 7.5% (20/265) Northeast, 11.9% (74/624) South, and 12.4% (65/526) West (6 missing location)).

The survey was completed by 192 professionals in planning/engineering (57.8%), public health (16.7%), law enforcement/EMS (8.9%), and other fields (16.7%) (Table 1). The length of time in the field varied widely across the sample. Professionals worked in all four census regions (39.8% South, 35.0% West, 14.5% Midwest, 10.8% Northeast) and census divisions.

**Table 1:** Descriptive characteristics and Vision Zero awareness among survey respondents (*n* = 192)

	Number	Percent	Missing
Field of work, primary			0
Planning/engineering	111	57.8	
Law enforcement/EMS	17	8.9	
Public health	32	16.7	
Other	32	16.7	
Length of time work in field			0
< 1 year	4	2.1	
1–5 years	35	18.2	
5–10 years	35	18.2	
10–15 years	37	19.3	
15–20 years	26	13.5	
20–25 years	19	9.9	
25–30 years	11	5.7	
More than 30 years	25	13.0	
Census region			6
Northeast	20	10.8	
Midwest	27	14.5	
South	74	39.8	
West	65	35.0	
Census division			6
New England	12	6.5	
Middle Atlantic	8	4.3	
East North Central	16	8.6	
West North Central	11	5.9	
South Atlantic	59	31.7	
East South Central	4	2.2	
West South Central	11	5.9	
Mountain	27	14.5	
Pacific	38	20.4	
Heard of Vision Zero			0
yes	174	90.6	
no	18	9.4	
Year heard of Vision Zero^a^			3
2012 or earlier	53	31.0	
2013	21	12.3	
2014	31	18.1	
2015	25	14.6	
2016	13	7.6	
2017	8	4.7	
I don’t know	20	11.7	
Does the municipality you work for have a Vision Zero campaign?^a^			4
Yes	71	41.8	
No	70	41.2	
I don’t know	29	17.1	
Are you involved in the Vision Zero campaign in the municipality where you work?^b^			0
Yes	39	54.9	
No	32	45.1	

^a^Only asked if participant answered “yes” that they had heard of Vision Zero

^b^Only asked if participant answered “yes” that their municipality has a Vision Zero campaign

Among the sample, 90.6% had heard of Vision Zero (Table 1). Awareness was higher in planning/engineering (97.3%), compared to law enforcement/EMS (76.5%) and public health (75.0%) (Table 2). Awareness was similar by number of years working in the field. Awareness was higher in the South (95.9%) and Northeast (95.0%) regions, followed by the West (90.8%) and Midwest (85.2%) regions, but the differences were not statistically significant.

**Table 2:** Participants awareness of Vision Zero by demographics (*n* = 192)

	Aware of Vision Zero (*n* = 174)		Not aware of Vision Zero (*n* = 18)		Missing	*p* value
	*n*	%	*n*	%		
Field of work, primary					0	0.0003
Planning/engineering	108	97.3	3	2.7		
Law enforcement/EMS	13	76.5	4	23.5		
Public health	24	75.0	8	25.0		
Length of time work in field					0	0.76
0–5 years	36	92.3	3	7.7		
5–15 years	66	91.7	6	8.3		
> =15 years	72	88.9	9	11.1		
Census region					6	0.28
Northeast	19	95.0	1	5.0		
Midwest	23	85.2	4	14.8		
South	71	95.9	3	4.1		
West	59	90.8	6	9.2		

*p* value from Pearson’s chi-squared test

Among those that heard of Vision Zero (*n* = 174), the year they learned of Vision Zero varied: 31.5% 2012 or earlier, 29.9% 2013–2014, 26.4% 2015–2016; 4.7% 2017, and 13.2% did not know. In the planning/engineering field, awareness generally occurred in earlier years (40.2% 2012 or earlier, 39.2% 2013–2014, 20.6% 2015–2017) (Table 3). However, for law enforcement/EMS and public health awareness was higher in more recent years. Generally, those who worked in the field longer heard about Vision Zero earlier. Participants working in the West census region generally learned about Vision Zero earlier in time, whereas the South region learned about Vision Zero more often in 2015–2017.

**Table 3:** Among participants aware of Vision Zero, responses to the year they first heard of it by demographics (*n* = 174)

	2012 or earlier		2013–2014		2015–2017		Missing	*p* value
	*n*	%	*n*	%	*n*	%		
Field of work, primary							0	0.0009
Planning/engineering	39	40.2	38	39.2	20	20.6		
Law enforcement/EMS	1	12.5	1	12.5	6	75.0		
Public health	2	10.5	5	26.3	12	63.2		
Length of time worked in field							0	0.05
0–5 years	6	17.1	13	37.1	16	45.7		
5–15 years	21	37.5	22	39.3	13	23.2		
> =15 years	26	43.3	17	28.3	17	28.3		
Census region							2	0.18
Northeast	7	38.9	7	38.9	4	22.2		
Midwest	6	31.6	6	31.6	7	36.8		
South	16	26.7	20	33.3	24	40.0		
West	24	46.2	19	36.5	9	17.3		

*p* value from Pearson’s chi-squared test

The sample size is 151, since 174 were aware of Vision Zero and 23 did not know the year they learned of it

Among those that heard of Vision Zero (*n* = 174), 41.8% worked at a municipality with a Vision Zero campaign, while 41.2% did not and 17.1% did not know. Among those working at a municipality with a Vision Zero campaign (*n* = 71), about half participated in the campaign (54.9%) while the other half did not (45.1%).

## Discussion

This survey of United States’ road safety professionals provides current information on the awareness of Vision Zero. The findings indicate that awareness of Vision Zero is high, particularly in planning and engineering fields. The awareness of Vision Zero to the fields of law enforcement/EMS and public health are reported to mostly occur in recent years, such that more promotion to those fields may be warranted. Awareness was lower in the Midwest, a location where Vision Zero uptake may be slower, and thus a region to target. Further promotion could happen through professional development avenues such as conferences, webinars, and social media with affiliate organizations. While this survey documented awareness of Vision Zero among road safety professionals, the awareness among non-professionals, such as those volunteering in local safety efforts, is not known. These individuals may be an ideal dissemination target as the road safety professional’s awareness becomes saturated.

Among the road safety professionals who completed the survey, almost half worked at a municipality with a Vision Zero campaign. These campaigns were located in all four census regions. This finding indicates both that Vision Zero is being implemented across the country and that dissemination is reaching professionals who are not associated with Vision Zero in their local communities.

The survey was grounded in the Diffusion of Innovations theory (Rogers, 2003), which identifies new ideas that are adopted early by innovators and early adopters. In this case, we assessed the rapidity of the diffusion of Vision Zero to United States’ safety professionals. The survey we used captured awareness of Vision Zero. There are several key characteristics that could speed the Diffusion of Innovations as applied to Vision Zero (Dearing et al., 2017). These include innovations that are less complex, easy to implement, flexible, produce observable changes, trialable (can be tried before committing to it), reversible (can be stopped if not working), and offer a relative advantage (the innovation is perceived as better than the idea before it). Integrating Vision Zero goals into other national, state, and local plans, such that it is compatible with other initiatives, is another way to support both dissemination and uptake. Moreover, identifying and highlighting the work of “opinion leader” organizations and municipalities can accelerate the uptake of such leaders’ practices and procedures (Dearing et al., 2017). These characteristics may be why Vision Zero awareness was so high.

These survey findings are subject to several limitations. First, the sampling strategy may not adequately represent all fields under study. The initial sample to draw from was lower for law enforcement/EMS, as well as the final response rate, and so estimates for this group may be less reliable. Second, the response rate was low, but not unexpected given the administration. Since we were unable to verify if an email was received and read, the true response rate is likely higher, but we cannot confirm this. Nevertheless, future efforts might address these limitations by conducting surveys in-person and offering an incentive to thank participants for their time. Verification of receipt of the emailed survey could also be tracked, to obtain a more accurate response rate. Third, for this type of study design, there is a threat of response bias, such that those who are aware of Vision Zero may be more likely to respond to the survey. However, on the survey invitation email, we did not mention Vision Zero. Fourth, while the survey was pilot tested, psychometric testing was not performed, such as assessing the test-retest reliability or validity of the items.

## Conclusions

Among professionals whose work involves understanding or improving the safety of people on roadways, awareness of Vision Zero is high. This is especially true among planning and engineering professionals, as well as those working in regions outside of the Midwest. Researchers and practitioners working in the broad field of road safety can help spread awareness of Vision Zero principles and identify best practices in order to rapidly diffuse them throughout professional and non-professional channels. Considering the Diffusion of Innovations (Dearing et al., 2017) as applied to Vision Zero, another next step is to evaluate how Vision Zero is being adopted, implemented, and maintained in communities, as well as the awareness and acceptability by community members. With widespread dissemination of the Vision Zero strategy to road safety professionals, another important step is to identify and disseminate the most promising policies and practices to share, in order to ultimately improve the safety of roadways for everyone.

## Additional file

Additional file 1: 2017 Roadway Safety Practitioner Survey. (PDF 25 kb)

## Abbreviation

EMS: Emergency medical services
