# Supplementary material for: Awareness of Vision Zero among United States’ road safety professionals
Source: Inj Epidemiol. 2018 May 8;5:21. doi: 10.1186/s40621-018-0151-1 (PMC5938221; doi:10.1186/s40621-018-0151-1)
Supplement: Supplementary file 1 — 2017 Roadway Safety Practitioner Survey. (PDF 25 kb) [file 40621_2018_151_MOESM1_ESM.pdf]

2017 Roadway Safety Practitioner Survey

Highway Safety Research Center at the University of North Carolina – Chapel Hill
Chapel Hill, North Carolina, United States

Q1.1 We invite you to complete this brief online survey about reducing fatal and serious injuries on roadways …

Q1.2 Will you be participating in our survey?
- ❍ I do not wish to answer this survey.
- ❍ I am willing to answer this survey.

Q2.1 Does your work involve understanding or improving the safety of people on roadways?
- ❍ Yes
- ❍ No

Q3.1 Do you work in any of the following fields: (mark all that apply)
- ❑ planning
- ❑ law enforcement
- ❑ engineering
- ❑ emergency management, such as emergency medical services (EMS)
- ❑ public health, such as injury prevention
- ❑ I work in another field ____________________

Q3.2 How long have you worked in this field?
- ❍ Less than 1 year
- ❍ 1 to 5 years
- ❍ 5 to 10 years
- ❍ 10 to 15 years
- ❍ 15 to 20 years
- ❍ 20 to 25 years
- ❍ 25 to 30 years
- ❍ More than 30 years

Q3.3 What is your title?

Q3.4 What is the name of the organization you work for?

Q3.5 Where is the organization located?

City

State

Q4.1 The next set of questions will ask you to identify individuals, organizations, and municipalities that work on reducing roadway fatalities and injuries. We might map the city you are from to the city of individuals, organizations, or municipalities that you recommend.

Q4.2 Please list up to three individuals outside of your workplace whose advice you seek or work you follow with respect to their work on reducing roadway fatalities and injuries. These individuals can work for any type of US organization, including governmental, nonprofit, or for-profit entities. For each individual please provide a name, organization, and city/state.

Q4.3 Individual 1
Name
Organization
City, State

Q4.4 Individual 2
Name
Organization
City, State

Q4.5 Individual 3
Name
Organization
City, State

Q4.6 Please list up to three organizations outside of your workplace whose example or reputation you follow with respect to their work on reducing roadway fatalities and injuries. These can include any type of US organization, including governmental, nonprofit, or for-profit entities. For each please provide the organization's name and city/state.

Q4.7 Organization 1
Organization Name
City, State

Q4.8 Organization 2
Organization Name
City, State

Q4.9 Organization 3
Organization Name
City, State

Q4.10 Please list up to three municipalities outside of your municipality whose example or reputation you follow with respect to their work on reducing roadway fatalities and injuries. These can include urban, suburban, and rural municipalities in the US. For each please provide the city and state.

Q4.11 Municipality 1
City, State

Q4.12 Municipality 2
City, State

Q4.13 Municipality 3
City, State

Q5.1 Have you heard of Vision Zero, a municipality-led "strategy to eliminate all traffic fatalities and severe injuries, while increasing safe, healthy, equitable mobility for all?"
- ❍ Yes
- ❍ No → skip to end of survey

Q5.2 In what year did you first hear about Vision Zero?
- ❍ 2012 or earlier
- ❍ 2013
- ❍ 2014
- ❍ 2015
- ❍ 2016
- ❍ 2017
- ❍ I don't know

Q5.3 Does the municipality where you work have a Vision Zero campaign?
- ❍ Yes
- ❍ No → skip to end of survey
- ❍ I don't know → skip to end of survey

Q5.4 Are you involved in the Vision Zero campaign in the municipality where you work?
- ❍ Yes (1)
- ❍ No (2)

<u>End of survey</u>: Thank you very much for your time. This concludes the survey.

<u>Acknowledgment</u>: This project was supported by the Collaborative Sciences Center for Road Safety (www.roadsafety.unc.edu), a United States Department of Transportation National University Transportation Center.
